# An Incidental Diagnosis of Conjoined Nerve Root After a Work-Related Back Injury: A Case Report

**DOI:** 10.7759/cureus.68436

**Published:** 2024-09-02

**Authors:** Christine L Zickler, Jose Martinez Elejalde, Amalia Landa-Galindez

**Affiliations:** 1 Occupational Health, Florida International University, Herbert Wertheim College of Medicine, Miami, USA; 2 Occupational Health, Baptist Health South Florida, Miami, USA; 3 Translational Medicine/Internal Medicine, Florida International University, Herbert Wertheim College of Medicine, Miami, USA

**Keywords:** lumbosacral spine, nerve entrapment, minimally invasive surgery, preoperative imaging, conjoined nerve root

## Abstract

Conjoined nerve roots (CNRs) are an uncommon condition often overlooked until surgery, posing significant intraoperative risks. This case report discusses a 21-year-old male diagnosed incidentally with a left lumbosacral CNR involving the fifth lumbar (L5) and first sacral (S1) spinal nerve roots following a work-related back injury, emphasizing the importance of preoperative imaging. Accurate early diagnosis of CNRs can prevent surgical complications and guide appropriate management, highlighting the need for careful preoperative planning and patient education.

## Introduction

Conjoined nerve roots (CNRs) are a rare nerve root anomaly found in 2-17.3% of people during lumbar spine imaging [1]. CNRs are defined as two adjacent nerve roots sharing a common dural sheath at some point along their path from the thecal sac [2]. They likely stem from abnormal root migration during embryological development and are often overlooked, even with advanced preoperative imaging techniques [2].

CNRs are most commonly located in the lumbosacral region, particularly at the fifth lumbar (L5) and first sacral (S1) vertebral segments. While they are typically unilateral, bilateral cases have been reported [3]. Clinical symptoms of conjoined nerve roots typically emerge after the age of 20, with no gender preference observed. Patients between the ages of 20 and 40 often report low back symptoms without radicular pain. In contrast, older patients frequently have a progressive course of sciatica. Patients typically complain of numbness and weakness, while physical examination findings sometimes show decreased sensation to pinprick in the corresponding dermatome [4]. However, CNRs can be asymptomatic and found incidentally on scans or during surgery [5].

CNRs are often undiagnosed until surgery. During spinal procedures that require nerve root mobilization, this can present considerable difficulties [6]. Thus, preoperative diagnosis of CNR is important to avoid damaging the nerve roots during surgery. It has been proposed that standard magnetic resonance imaging (MRI) and computed tomography (CT) scans can aid in the diagnosis of CNR preoperatively [7].

In this report, we present a case of a conjoined left L5-S1 nerve root found incidentally in a 21-year-old after a work-related back injury. This case aims to highlight the importance of preoperative imaging to avoid damaging the nerve roots intraoperatively.

## Case presentation

A 21-year-old male presented to occupational health with upper and lower back pain two days after catching a patient who was about to fall due to a syncopal episode. The patient described the pain as soreness, radiating throughout his entire spine. He denied any numbness or tingling in his lower and upper back. He described a previous traumatic injury to his upper back five years ago while running track. When assessing the patient, he rated it as a 3 out of 10. On physical examination, there was no point tenderness of the thoracolumbar spine or paraspinal muscles. There was tenderness of the lumbosacral region with lumbar flexion and extension but no tenderness with lateral flexion or rotation. He had a full range of motion and strength in all extremities, no pain with ambulation, and normal reflexes.

**Figure 1:**
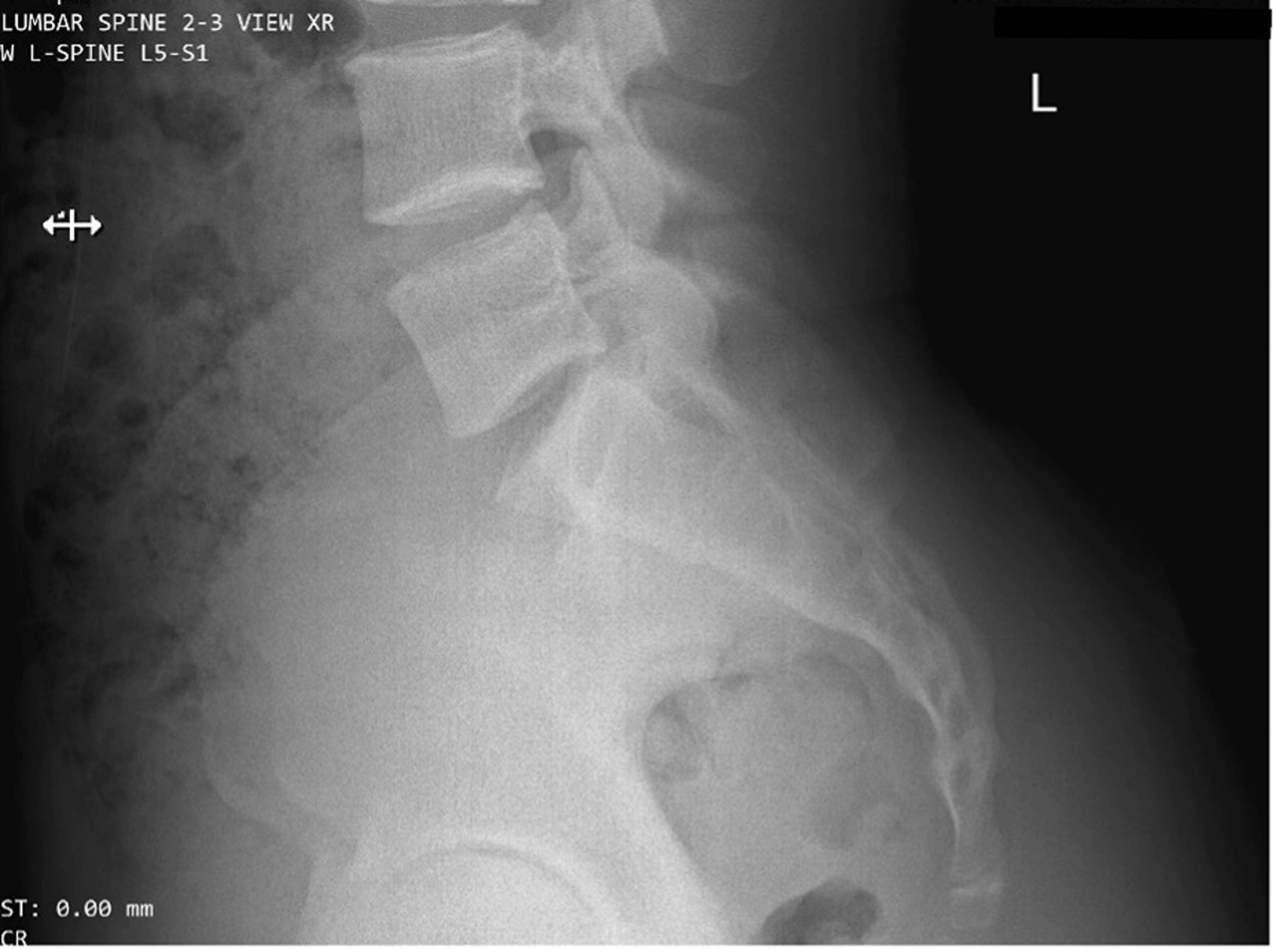
Lumbosacral spine lateral view plain films read as unremarkable for this patient, with the fifth lumbar-first sacral (L5-S1) conjoined nerve roots not readily identifiable.

On the day of the injury, the patient presented to the emergency department where lumbar spine plain radiographs were performed. Radiology interpreted the radiographs as showing an unremarkable lumbar spine with no acute abnormalities (see Figure 1). He was diagnosed with muscle strain and prescribed ibuprofen 600 mg twice daily as needed.

Four days after the injury, the patient presented to occupational health for follow-up. He was still symptomatic, with pain 3 out of 10. He stated the pain improved when lying down but was aggravated by sitting/standing. Physical examination findings were unchanged compared to the prior occupational health visit. Due to persistent pain, MRIs without contrast of the thoracic and lumbar spine were ordered.

**Figure 2:**
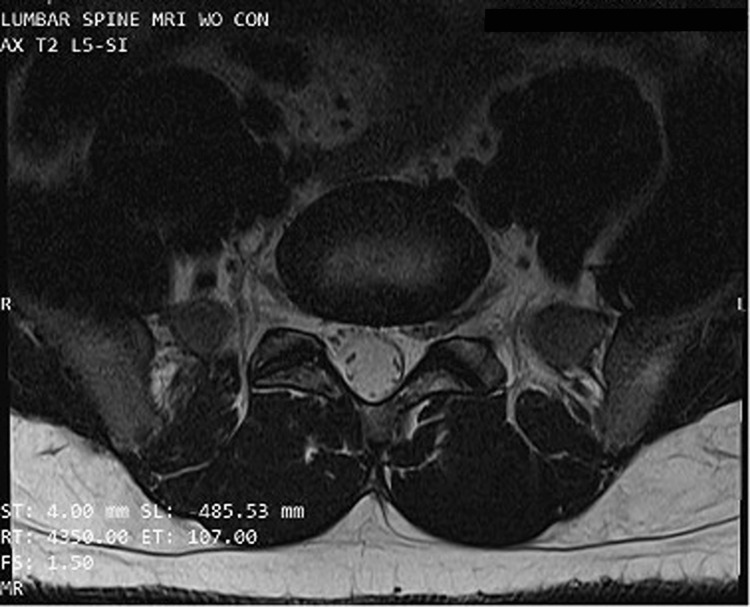
Axial magnetic resonance imaging view at the level of the fifth lumbar/first sacral (L5-S1) vertebrae reveals asymmetry of the left and right nerve root exit into the foramina.

Thoracic spine MRI showed a small central herniation between the fifth and sixth thoracic vertebrae (T5-6), and a left paramedian herniation and annular fissure between the ninth and tenth thoracic vertebrae (T9-10). Lumbar MRI showed disk bulges between lumbar vertebrae three to four (L3-4), lumbar vertebrae four to five (L4-5), and lumbar vertebrae five to sacral vertebra one (L5-S1), along with bilateral facet mild hypertrophy in L5-S1. An incidental left L5-S1 CNR was discovered (see Figure 2).

The patient followed up with occupational health four days later. He was feeling better, with less back discomfort. The pain was located across the right upper, mid, and lower back region, again aggravated by positional changes. He denied numbness/tingling/weakness and had no limitations. At the time of this appointment, he was working light duty without difficulties.

A recommendation was made for physical therapy biweekly for four weeks. The patient was referred to physiatry for management and follow-up of his back pain. He was advised to follow up with his personal primary care provider for the incidental finding of congenital left L5-S1 CNR.

## Discussion

Although CNRs are well-represented in the literature, they are still commonly undiagnosed until surgery. As CNRs can pose danger intraoperatively, such as a considerable risk of neurologic injury when nerve root mobilization is required during spinal procedures, preoperative diagnosis is crucial [7].

The young patient in our case would not have known about his CNR if he had not presented to occupational health for a follow-up of an incidental work-related injury. He had a history of prior back injuries, first injuring his back while running track in high school. It is possible that this patient would have required a spinal procedure as an adult due to his recurring injuries. Fortunately, he had this congenital anomaly identified at a young age, preventing potential future surgical complications.

Minimally invasive surgery (MIS) is commonly performed in the treatment of lumbosacral nerve entrapment disorders. However, for individuals with a CNR, the limited visualization during MIS increases the risk of devastating nerve root injuries [8]. Careful preoperative planning is required for patients with CNR anomalies. Classic MRI findings may include asymmetry of the anterolateral corners of the dural sac (corner sign), extradural fat between the CNR sleeve and asymmetric dura (fat crescent sign), and parallel track of the affected nerve root at the affected level (parallel sign) [3].

Our patient did not require surgery but is at risk for future complications. Traumatic injuries have been reported to provoke intense radicular pain in individuals with a CNR and may lead to severe disabilities [4]. Although our patient had no complaints of radicular pain and was referred to physical therapy for this work-related injury, close follow-up was advised and he was cautioned about high-impact contact sports/lifting.

However, due to the financial costs and time involved, initial follow-ups are often inaccurate, leading to the choice of the quickest and most convenient method. Additionally, because this type of disease is rare, healthcare systems typically do not allocate the necessary funds to identify such cases. Therefore, the cost-benefit aspect should be addressed in future studies.

## Conclusions

Undiagnosed CNRs can cause significant intraoperative complications. Avoiding long-term complications from a CNR is possible, but timely diagnosis and appropriate care measures need to be prioritized. Based on the analysis, while medical management was the primary focus of treatment in this case, patient education remains a key factor in long-term management. Furthermore, conservative non-surgical management should be considered as a first-line approach when appropriate. Future studies should investigate the cost-benefit of identifying these cases.
